# Bioactivity and Toxicity of *Senna cana* and *Senna pendula* Extracts

**DOI:** 10.1155/2018/8074306

**Published:** 2018-04-02

**Authors:** J. A. Monteiro, J. M. Ferreira Júnior, I. R. Oliveira, F. L. A. Batista, C. C. C. Pinto, A. A. S. Silva, S. M. Morais, M. G. V. Silva

**Affiliations:** ^1^Department of Organic and Inorganic Chemistry, Federal University of Ceará, Pici Campus, 60021-970 Fortaleza, CE, Brazil; ^2^Faculty of Education, Sciences, and Letters of Inhamuns, Ceará State University, CECITEC, Street Sólon Medeiros S/N, 60.714-903 Tauá, CE, Brazil; ^3^Ceará State University, Av. Dr. Silas Munguba 1700, 60714-903 Fortaleza, CE, Brazil; ^4^Department of Physiology and Pharmacology, Federal University of Ceará, Av. Cel. Nunes de Melo 1127, 60430-270 Fortaleza, CE, Brazil

## Abstract

This work investigated the content of total polyphenolic compounds and flavonoids as well as their toxicity and larvicidal and acetylcholinesterase inhibitory activities. The antioxidant activities of two medicinal *Senna* species extracts (*Senna cana* and *Senna pendula*) were also investigated. The ethanol extract of the leaves of *S. cana* and the ethanol extract of the branches of *S. pendula* presented the best performance in the DPPH/FRAP and ABTS/ORAC assays, respectively. For the inhibition of acetylcholinesterase, the hexane extract of the flowers of *S. pendula* presented the lowest IC_50_ value among the ethanol extracts of the leaves of *S. cana* and showed the best performance in some assays. The hexane extract of the leaves of *S. pendula* and the hexane extract of the branches of *S. cana* were moderate to *Artemia salina* Leach. In the quantification of phenols and flavonoids, the ethanol extract of the leaves of *S. cana* presented the best results. The ethanol extracts of the leaves of *S. cana* were found to be rich in antioxidants, phenolic compounds, and flavonoids. These results indicate the antioxidant potential of the extracts of *Senna* species and can be responsible for some of the therapeutic uses of these plants.

## 1. Introduction

The genus *Senna* (Fabaceae) includes about 260 species, 200 of which occur in the Americas. Several of these occur in the Brazilian northeastern semiarid region, such as *S. martiana* and *S. spectabilis* var. *excelsa*, which are used in folk medicine to treat colds, as laxatives, and for antioxidant, cytotoxic, and acetylcholinesterase inhibitory activities [1].

Many of these species are reported in the literature to contain anthraquinone glycosides, responsible for laxative activity, and polyphenolic metabolites such as flavonoids, which are scientifically recognized as having considerable leishmanicidal and antioxidant activity, among other biological activities [1–3].

**Figure 1:**
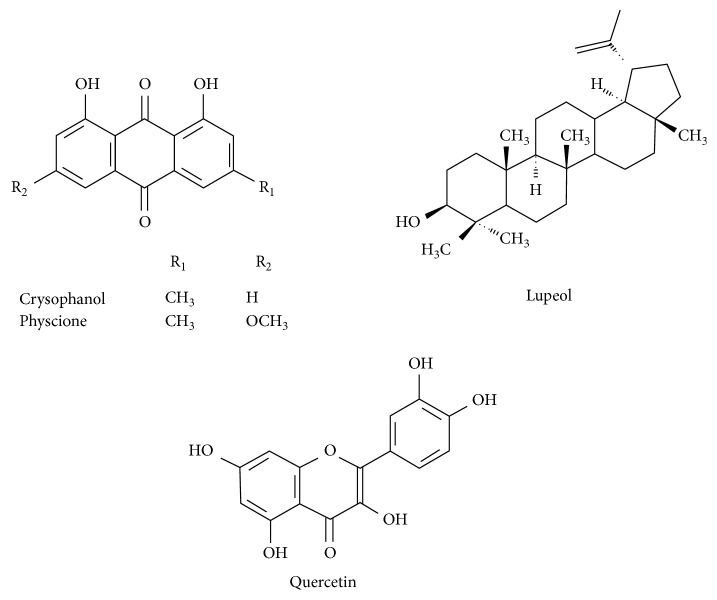
Structural representation of chemical compounds of *Senna* species.

Anthraquinones such as chrysophanol (1,8-dihydroxy-3-methylanthraquinone) and physcione (1,8-dihydroxy-3-methyl-6-methoxy-anthraquinone) (Figure 1), triterpenes such as lupeol (*β-*lup-20 (29)-en-3-ol), and flavonoids such as quercetin are commonly identified in various species of *Senna*. These compounds have been associated with anti-inflammatory, antimicrobial, antitumor, antimalarial, cardioprotective, and antioxidant activities and are also used to treat liver disease and psoriasis [4–9].

Aiming to minimize the use of animals in experiments, many companies producing natural products already use the plant toxicity test against *Artemia salina* in more advanced research to find bioactive compounds. This method is efficient, relatively fast, inexpensive, and requires small sample amounts and no great asepsis care [10].

With the current spread of diseases caused by arboviruses transmitted through *Aedes aegypti* mosquitoes, such as dengue, zika, and chikungunya, the search for new sources of natural repellents extracted from plants is very important to control outbreaks [11].

Therefore, the aim of this study was to evaluate the pharmacological potential of *S. cana* HS Irwin and Barneby and *S. pendula* HS Irwin and Barneby, native species of the Brazilian northeastern, through different methods of determining antioxidant activity, toxicity, larvicidal activity, acetylcholinesterase inhibition, and flavonoid content (Supplementary Materials (available here)).

## 2. Materials and Methods

### 2.1. Preparation of Plant Extracts

**Figure 2:**
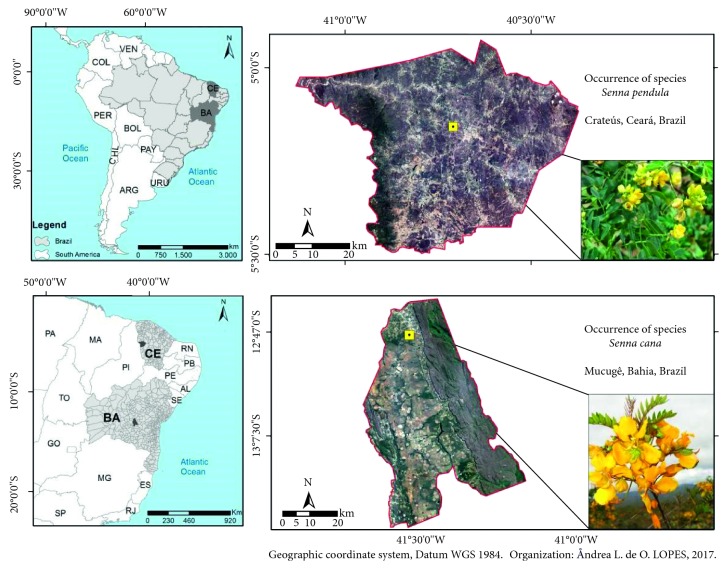
Map of plant collection showing sites from Bahia and Ceará State, Brazil.

The leaves and branches of *Senna cana* were collected in Mucugê, Bahia State (−12° 57′ 7900″ S and −41° 19′ 3600″ W, elevation of 400 m). The leaves, branches, and flowers of *S. pendula* were collected in Cratéus, Ceará State (−5° 17′ 833″ S and −40° 67′ 75″ W, elevation of 300 m) (Figure 2). Both places are in northeastern Brazil. The botanical material was deposited in the Prisco Bezerra Herbarium of Universidad Federal of Ceará with respective identification numbers of 50297 and 54075.

The plant material was dried, weighed, and macerated thoroughly in *n*-hexane for seven days at room temperature. Afterwards, the hexane extract was filtered and concentrated in a rotary evaporator, obtaining the hexane extracts. This process was repeated with ethanol to obtain the ethanol extracts. The residues were dried and stored at 27°C. The extracts obtained are shown in Table 1.

### 2.2. Chemical Screening

The tests were carried out according to the method proposed by Matos [12], using freeze-dried extracts. The main secondary metabolite classes present in the extracts were identified by chemical reactions with specific reagents and formation of precipitates or color changes. Some of the chemical tests performed are described below.

#### 2.2.1. Test for Detection of Xanthones and Flavonoids

Solutions of the extracts were prepared at a concentration of 5 mg·mL^1^ in methanol. Aliquots of 5 mL of this solution were removed and placed in test tubes for chemical testing. Magnesium strips and 4 drops of concentrated HCL were added to the tubes. The presence of flavonoids and xanthones was detected by the appearance and intensification of the red color in the solution.

#### 2.2.2. Test for Detection of Tannins

Solutions of the extracts were prepared in the same way as for the flavonoid test (5 mg·mL^1^ in methanol). Aliquots of 5 mL of this solution were removed and placed in test tubes for chemical testing. Distilled water (5 mL) was added, and the solution was filtered to remove any solids, after which 5 drops of FeCl_3_ were added to attain a concentration of 10%. The formation of blue color indicates the presence of hydrolyzable tannins and green color indicates the presence of condensed tannins.

#### 2.2.3. Test for Detection of Anthraquinones

Solutions of the extracts were prepared (5 mg·mL^1^ in methanol), and 5 mL aliquots of this solution were removed and placed in test tubes for chemical testing, with addition of chloroform (5 mL) under stirring. After 15 minutes, the chloroform phase was collected and 1 mL of 5% NaOH was added. Purple color indicates the presence of quinones.

#### 2.2.4. Test for Detection of Triterpenoids and Steroids

Ten milligrams of the dry extract was solubilized with 6 mL of chloroform. The solution was filtered, and 1 mL of acetic anhydride was added, followed by 3 drops of concentrated H_2_SO_4_ under slow stirring. A bluish-green color indicates the presence of the free triterpenoids.

### 2.3. Antioxidant Activity

#### 2.3.1. DPPH Radical Scavenging Assay

In the spectrophotometric procedure, 3.9 mL of a methanol solution of DPPH (2,2′-diphenyl-1-picrylhydrazyl) at 6.5 × 10^−5^ mol·L^−1^ and 0.1 mL of methanol solutions of extracts or positive control, di-tert-butylmethylphenol (BHT), were mixed, and the absorbance of the reaction was read at 515 nm. The test was performed in triplicate at various concentrations (mg·mL^−1^) [13]. Absorbance measurements were determined in a Spekol 1100 spectrophotometer.

#### 2.3.2. Folin–Ciocalteu Method

Total phenol content was determined by the spectrophotometric method using the Folin–Ciocalteu reagent and gallic acid as the reference standard [14]. Ethanol extracts (7.5 mg) were dissolved in methanol and transferred to a 25 mL volumetric flask, and the final volume was completed with methanol. An aliquot of 100 *µ*L of the latter solution was shaken with 500 *µ*L of the Folin–Ciocalteu reagent and 6 mL of distilled water for 1 min. After this time, 2 mL of 15% Na_2_CO_3_ was added to the mixture and stirred for 30 s. Finally, the solution was diluted to 10 mL volume with distilled water. After 2 h of incubation, the absorbance of the samples was measured at 750 nm.

#### 2.3.3. ABTS Radical Scavenging Assay

This method was based on Re et al. [15]. It measures the antioxidant capacity based on the ability of the substances to inactivate the cation radical 2,2′-azinobis-(ethylbenzo-thiazoline-6-sulfonic acid) diammonium salt (ABTS°^+^). Solutions of the extracts were prepared at a concentration of 600 mg·L^−1^. Aliquots of 10 *µ*L, 20 *µ*L, and 30 *µ*L of these solutions were added to test tubes, and the volume in the first two cases was completed with distilled water to 30 *µ*L (extract and water). In dark environment, 3 mL of each solution (radical ABTS°^+^ + ethanol P.A.) was added to a test tube, which already had absorbance preset to 0.70 in the absence of light. Readings were taken at 734 nm in a spectrophotometer six minutes after addition of the radical. The percentage inhibition of ABTS°^+^ was determined from the standard curve of trolox, and the results were expressed as TEAC (trolox equivalent antioxidant capacity) *µ*mol·g^−1^.

#### 2.3.4. Potential Antioxidant FRAP (Ferric-Reducing Antioxidant Power)

This method is based on the capacity of metabolites to reduce Fe^3+^ to Fe^2+^. When this occurs in the presence of 2,4,6-tripyridyl-S-triazine (TPTZ), the formation of Fe^3+^/TPTZ with blue staining of the Fe^2+^ occurs.

The extracts were prepared in methanol at different concentrations between 0.25 and 1.0 mg/mL. Ten microliters of the extracts was first incubated with 30 *µ*L of bidistilled water and 300 *µ*L of FRAP reagent, consisting of 25 mL of acetate buffer (300 mmol·L^−1^ sodium acetate, pH = 3.6), 2.5 mL of TPTZ (TPTZ in 10 mmol·L^−1^ and HCl 40 mmol·L^−1^), and 2.5 mL of FeCl_3_ at 37°C, before measurement. A ferrous sulfate calibration curve (0.01–1.0 mmol·L^−1^) was used, and the results were expressed as (Fe^2+^ mmol·L^−1^)·L^−1^. The reaction was measured at 595 nm in a universal microplate reader (ELx 800, BioTek Instruments, Winooski, Vermont, USA) after 5 min resting time. Using these regression curves, the EC values were calculated as the concentrations of the antioxidant (expressed as mg·mL^−1^) giving an absorbance equivalent to a 1 mM Fe(II) solution according to Pulido et al. [16].

#### 2.3.5. Potential Antioxidant ORAC (Oxygen Radical Absorbance Capacity)

This assay measures the capacity of the antioxidant to sequester the peroxyl radicals that are generated by a free radical source, a small molecule called 2,2′-azobis-(2-amidinopropane) dihydrochloride (AAPH), using fluorescein (0.21 *μ*mol·L^−1^ of ORAC buffer) as a redox indicator [17].

From a stock solution of DMSO, trolox (10 mmol·L^−1^) was diluted in ORAC buffer solution to a concentration of 20 *μ*mol·L^−1^. The ORAC buffer contains 75 mmol·L^−1^ of sodium hydrogen phosphate/hydrogen phosphate potassium with pH = 7.4.

The decline of fluorescein was measured at 37°C at constant intervals of 2 minutes between measurements until completing 122 min using a CytoFluor 4000 fluorescence microplate reader (excitation wavelength at 530 nm was measured every minute for 25 min and emission wavelength at 585 nm was measured every minute for 30 min) (Perspective Biosystems, Minnesota, USA).

The final ORAC results were calculated using a regression equation between the trolox concentrations and AUC, expressed as ORAC units, where 1 ORAC unit inhibits the decline produced by 1 *μ*mol·L^−1^ of trolox.

### 2.4. Total Flavonoid Contents

The total flavonoid contents of the extracts were determined using the spectrophotometric method described in Quettier et al. [18], where quercetin was used as a standard, in a solution of aluminum chloride. Twenty milligrams of each extract was placed in a test tube, and methanol was added to complete the volume of 50 mL, producing a second extract. Then, 5 mL of this second extract was removed and 0.5 mL of 2% AlCl_3_ solution was added, after which the volume was completed to 10 ml with 5% acetic acid solution. Following incubation for 30 min, the absorbance of the reaction mixture was measured at *λ*_max_ = 425 nm with a Femto 700 plus spectrophotometer.

The calibration curve was plotted using concentrations of 5, 10, 25, 50, and 75 *µ*g·mL^−1^ of quercetin.

### 2.5. Evaluation of Antiacetylcholinesterase Activity

#### 2.5.1. Qualitative Analysis

A 5 *µ*L aliquot of the extract (10 mg·mL^−1^) was placed on a thin-layer chromatography plate. Then, a mixture of (1 : 1) acetylcholine iodide (ATCI) mmol·L^−1^ with Ellman's reagent (5,5′-dithiobis-[2-nitrobenzoic acid] (DTNB); 1 mmol·L^−1^) was sprayed on the plate, followed by spaying with acetylcholinesterase. After 10 minutes, if a yellow color appears on the inhibition of the enzyme, a white halo is formed around the “spots” where samples were applied. Eserine salt was used as the standard (2 mg/mL) [19].

#### 2.5.2. Quantitative Analysis

25 *μ*L of acetylthiocholine iodide (15 mmol·L^−1^), 125 *μ*L of 5,5′-dithiobis-[2-nitrobenzoic acid] in Tris/HCL solution (50 mmol·L^−1^, pH = 8, with 0.1 mmol·L^−1^ NaCl and 0.02 mmol·L^−1^ MgCl_2_.6H_2_O (3 mmol·L^−1^, DTNB or Ellman's reagent)), 50 *μ*L of Tris/HCL solution (50 nmol·L^−1^, pH = 8.1% bovine serum albumin (BSA)), and 25 *μ*L of the extract dissolved in ethyl acetate and diluted 10-fold in a Tris/HCL solution (50 mmol·L^−1^, pH = 8) were used to obtain a final concentration of mg·mL^−1^. The absorbance was measured at a wavelength of 405 nm for 30 seconds. Immediately afterwards, 25 *μ*L of the enzyme acetylcholinesterase (0.25 U·mL^−1^) was added, and the absorbance was read every minute for 25 minutes. Eserine salt was used as the positive standard, and the dilutions of both the samples and the standard were based on a concentration of 20 mg·mL^−1^ [19, 20].

### 2.6. Toxicity against *Artemia salina*

For these analyses, the *A. salina* eggs were incubated in artificial seawater at room temperature for 48 hours in a microaquarium. The extracts were dissolved in DMSO and saline water in different concentrations. The tests were performed in triplicate. After 24 h of exposure, the number of dead larvae of the samples was recorded and the concentration necessary to kill 50% of the larvae (LC_50_) was calculated. The potential toxicity (PT) of the samples was classified as (A) nontoxic (LC_50_ > 1000 *μ*g·mL^−1^); (B) low toxicity (500 < LC_50_ ≤ 1000 *μ*g·mL^−1^); (C) moderate toxicity (100 < LC_50_ ≤ 500 *μ*g·mL^−1^); and (D) high toxicity (LC_50_ < 100 *μ*g·mL^−1^) [21].

### 2.7. Evaluation of Larvicidal Activity

This assay was based on Cavalcanti et al. [22]. Solutions of 100, 250, 500, and 1000 *µ*g·mL^−1^ of all extracts were prepared with water and DMSO. Then, 25 *Aedes aegypti* larvae were added in stage 3. The solution was left with the larvae for 24 hours; then, the dead larvae were counted and the LC_50_ of each extract was calculated.

### 2.8. Statistical Analysis

The relation between the phenolic compound and antioxidant variables was determined by linear regression using Excel and Origin 6.0 software.

## 3. Results and Discussion

### 3.1. Chemical Screening

The chemical screening of the hexane extracts LHESC, BHESC, LHESP, BHESP, and FHESP was positive only for anthraquinones and triterpenes. However, for the ethanol extracts LEESC, BEESC, LEESP, BEESP, and FEESP, the presence of anthraquinones, flavonoids, triterpenes, tannins, and xanthones was confirmed. The results for the chemical screening of ethanol extracts are shown in Table 2. These metabolites are frequently found in *Senna* species [23].

### 3.2. Determination of Antioxidant Activity and Quantification of Flavonoids and Total Phenol Contents

Antioxidant tests can be divided into two types: the direct method (ORAC), which is used to ascertain chemical kinetics, and indirect methods (DPPH, ABTS, and FRAP), which are mediated by electron transfer. Each method has its specificity. Some methods involve an acid medium, as is the case of the FRAP assay, while others are already in the basic medium, such as the ABTS assay, and other tests are carried out in a neutral medium, such as the test of total phenolic content. The pH of the medium can have an important effect on antioxidant capacity of the compounds [17].

In this work, we related the results to ethanol extracts because the hexane extracts were negative for flavonoids, which are recognized as having a significant antioxidant potential. Additionally, the hexane extracts were not compatible with all the antioxidant screening methods. The results obtained for the antioxidant tests are shown in Table 3.

The extracts that showed the best performance against DPPH/FRAP were the ethanol extract of the leaves of *S. cana* (LEESC) (IC_50_, 59.5 *μ*g·mL^−1^, and EC, 304.8 mg·mL^−1^) and the ethanol extract of the branches of *S. pendula* (BEESP) (IC_50,_ 62.1 *μ*g·mL^−1^, and EC, 329.17 mg·mL^−1^). For the ABTS/ORAC assays, the best results were obtained for the same samples of the two previous tests, but an inversion occurred between the first and second places, where the ethanol extract of the branches of *S. pendula* (BEESP) (4886.7 *μ*mol TE·g ^−1^ and 5.01 units) showed the best performance, followed by the ethanol extract of the leaves of *S. cana* (LEESC) (4440.4 *μ*mol TE·g^−1^ and 4.72 units).

The extract that presented the worst performance in all the antioxidant trials was the extract of the branches of *S. cana* (BEESC), in contrast to the excellent performance presented by the leaves of *S. cana* (LEESC). This indicates that the contents and classes of compounds found can vary sharply with the plant part.

According to the literature, direct methods (in this case, ORAC) are more suitable for the evaluation of antioxidant activity, especially those based on the controlled chain reaction model, because in general they are more sensitive. The best practice is always to make a comparison between the data obtained by both methods (direct and indirect) in order to obtain greater analytical safety [17, 24].

#### 3.2.1. Correlation between the Antioxidant Assays

**Figure 3:**
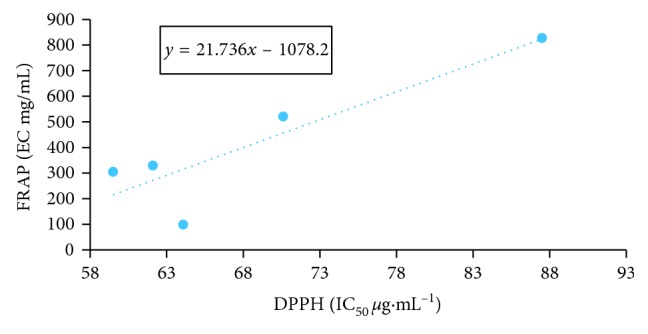
Correlation between DPPH and FRAP assays.

**Figure 4:**
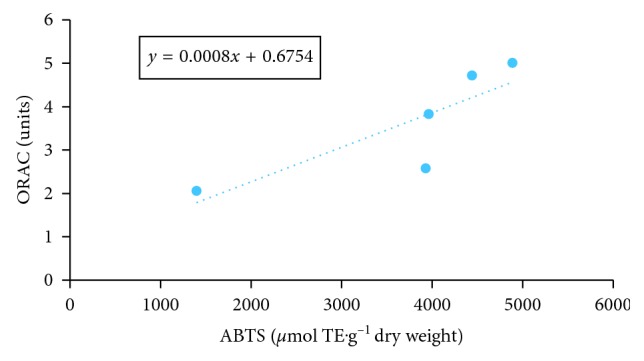
Correlation between ABTS and ORAC assays.

According to the statistical analysis and a comparison of correlation between the 4 tests, the strongest correlation (Table 4) among the antioxidant capacity assays was observed between the DPPH and the FRAP assays (Figure 3) (*r*=0.8908; *n*=5) and the ABTS and ORAC assays (Figure 4) (*r*=0.8353; *n*=5).

In the spectrophotometric quantification of the total flavonoid content (Table 5), the ethanol extract of the leaves of *S. cana* (LEESC) presented the best results (228.9 mg·g^−1^), followed by the ethanol extract of the leaves of *S. pendula* (LEESP), which presented a value of 221.1 mg·g^−1^.

Regarding the total phenolic content, the leaf extract of *S. cana* (LEESC) also presented the best result (724.5 mg EAG·g^−1^ extract), which confirms that this extract, because it contains a high phenolic content, automatically has a high content of antioxidants, since phenolic compounds are excellent natural antioxidants. It is noteworthy that the extracts that presented the best results had more satisfactory performance than the standards used (BHT and rutin), even though rutin is a flavonoid, which is a natural antioxidant. This suggests that the synergism of the compounds present in extracts may have influenced this excellent result. Phenolic compounds have been reported in the literature to have good antioxidant activity [25, 26].

Like in the antioxidant tests, the branch extract of *S. cana* (BEESC) showed the lowest phenolic content and the second lowest total flavonoid content. These results partly explain the weak performance of this extract in the antioxidant trials because of the lower content of phenolic compounds and flavonoids in this extract [7, 9].

Another factor that can be considered for the good performance of the leaf extracts of *S. cana*, both in relation to the different antioxidant tests and the total phenolic content, may be the high content of flavonoids present in the extract, considering that the extract was prepared in an organic solvent, which increases the solubility of these flavonoids in the solution. While ORAC and Folin–Ciocalteu tests are not suitable for measuring liposoluble antioxidants, ABTS can measure the activity of both water-soluble antioxidants and liposoluble ones and DPPH in turn is soluble only in organic solvents [27, 28].

When comparing the value obtained with the values reported in the literature, the TEAC value found for the ethanol extract of the leaves of *Senna alata* was 125 *μ*mol trolox·g^−1^ (31.29 mg trolox·g^−1^), a value well below the values obtained for all extracts studied in this work [29]. According to Liczano [7], the aqueous extract of the leaves of *S. reticulata* presented an ORAC value of 226.6 *μ*mol trolox·g^−1^ and a TEAC value of 34.04 *μ*mol trolox·g^−1^. Both these results are lower than those found for all extracts presented here. Based on the data observed, the extracts of *S. cana* and *S. pendula* had excellent performance in the different antioxidant trials and contain high concentrations of phenolic compounds and flavonoids in their composition.

Also in comparison with the values in the literature, Mak et al. [30] reported that the ethanol extract of the flowers of *S. bicapsularis* L. contained total flavonoids and phenols of 12.93 mg quercetin·g^−1^ extract and 262.23 mg EAG·g^−1^ extract, respectively. In contrast, the extracts of *S. cana* and *S. pendula* studied contained much higher total flavonoids and phenols, and the lowest value found for the flavonoid content was for the ethanol extract of the branches of *S. pendula*, which presented a value of 87.04 mg of quercetin·g^−1^ extract. For the total phenol content, the lowest value was found for the ethanol extract of the branches of *S. cana*, which presented a content of 473.7 mg EAG·g^−1^ extract.

#### 3.2.2. Correlation between Total Phenolic Content and Antioxidant Capacity

The best correlation (Table 6), that is, positive correlation, between total phenolic compounds and antioxidant capacity was obtained in the ABTS assay (*r*=0.595982; *n*=5) followed by the ORAC assay (*r*=0.576957; *n*=5).

#### 3.2.3. Correlation between Total Flavonoid Content and Antioxidant Capacity

The best correlation (Table 7) between total flavonoid and antioxidant capacity was obtained in the ABTS assay (*r*=0.29654; *n*=5) followed by the ORAC assay (*r*=0.28349; *n*=5).

### 3.3. Evaluation of Antiacetylcholinesterase Activity

Plants are promising sources of new drugs, including some used to treat Alzheimer's disease (AD), such as galantamine. The drugs available at present are effective only in the early stages of AD, whose period is very short. Therefore, it is important to search for new drugs of natural origin that inhibit the enzyme acetylcholinesterase (AchE), both in the early stages and advanced stages of AD [31, 32].

Phenols and flavonoids are important natural products that inhibit acetylcholinesterase and thus restore the level of acetylcholine, which is essential for brain function [33]. Since the *Senna* extracts contained significant concentrations of phenolic compounds and flavonoids, the evaluation of antiacetylcholinesterase activity was carried out to confirm this aspect.

To date, no studies have been published evaluating the anticholinesterase activity of extracts of *Senna* species. There are only two reports involving alkaloids isolated from *Senna multijuga* who had this activity [34, 35].

The results obtained for the inhibition of cholinesterase are shown in Table 8. Only the ethanol extracts of the branches and leaves of *S. cana* showed no activity in this qualitative test. The hexane extract of the bark of *S. pendula* was the only one that presented significant activity.

The hexane extracts of *S. cana* and the hexane and ethanol extracts of *S. pendula* presented inhibitory activity and can be considered for future studies against Alzheimer's disease, neurodegenerative diseases, and dysfunction of the cholinergic system.

Through quantitative analysis of anticholinesterase activity, it was possible to verify an IC_50_ value different from the value obtained for the eserine standard. The extract that presented the best IC_50_ value was FHESP, in agreement with the result obtained in the qualitative test, where this extract also presented the best performance.

Another observation is related to the ethanol extracts of *S. cana,* which in the qualitative test had a negative result, while in the quantitative test, they presented satisfactory results. The LEESC extract presented the third best performance in the quantitative test. This fact may be related to the high content of phenolic compounds and flavonoids found in this extract. Regarding antioxidant activity, this same extract performed best in the DPPH and FRAP assays and the second best in the ABTS and ORAC assays. These results corroborate the finding of Penido et al. [33] that the higher the content of phenolic compounds and flavonoids, the better the performance against inhibition of acetylcholinesterase.

### 3.4. Evaluation of Toxicity against *Artemia salina*

According to Silva et al. [36], the determination of the toxicity of plant samples against *Artemia salina* L. allows the evaluation of toxicity involving only one parameter: life or death. Therefore, this model is considered as a preliminary form of testing, with low cost and easy handling, to identify bioactive compounds. The absence of cytotoxicity of the extracts tested against *A. salina* is an indicator that the plant can be well tolerated by the biological system.

In the *in vitro* evaluation of the toxicity of the samples against *A. salina*, only the BHESC and LHESP extracts were able to cause 50% mortality of the larvae of *A. salina*, and they also presented low toxicity, with LC_50_ of 790.94 and 746.35 *μ*g·mL^−1^, respectively. All values are reported in Table 9.

These results confirm the toxic action for only these extracts, since only the samples with LC_50_ less than 1000 *μ*g·mL^−1^ are considered to be toxic according to the classification described by Meyer [21]. These results corroborate the possible use of the other extracts tested, as reported by Simões and De Almeida [37]. If a sample is not shown to be toxic to *A. salina*, its effects will also be the same in humans.

The toxicity results of the BHESC and LHESP extracts are also valuable, since samples that are toxic to *A. salina* can contain bioactive compounds with antitumor, antimalarial, trypanosomicidal, and insecticidal potential [38], possible antiplasmodic activity [39], and antimicrobial effect [40].

Parra et al. [41], when testing the aqueous extract of the leaves of *Senna alata*, obtained LC_50_ of 7.74 *μ*g·mL^−1^, much lower than what is considered to be below toxicity. In other words, this aqueous extract showed high toxicity, unlike the ethanol extracts of *S. cana* and *S. pendula* shown in this work.

### 3.5. Evaluation of Larvicidal Activity

The results of the larvicidal activity were analyzed and interpreted according to LC_50_ values. Due to the low solubility of the hexane extracts in DMSO, only the ethanol extracts of each part of the two *Senna* species were used. According to the literature, for results to be considered good, the sample should have LC_50_ below 100 ppm. As seen in Table 10, all the results found for *S. cana* and *S. pendula* were well above 100 ppm, meaning that these plants are not considered promising sources of substances with relevant larvicidal activity [22].

Edwin et al. [42] investigated the larvicidal activity of the ethanol and aqueous extracts of leaves and stems of *Senna alata*: for the aqueous extract, the values were 0.840 (% w/v) for the leaves and 0.935 (% w/v) for the stems, while for the ethanol extract of the leaves, the value was 0.791 (% w/v) and for the stems, it was 0.923 (% w/v). Unlike the extracts of *S. cana* and *S. pendula*, those of *S. alata* [43] and *S. occidentalis* [23] presented excellent larvicidal activity. When comparing the chemical composition of these species of *Senna*, there is a very peculiar difference, which may explain this great difference in toxicity against *Aedes aegypti*: *S. alata* was found to contain cassiaindoline, a dimeric indole alkaloid, a compound that has not been identified in *S. cana* and *S. pendula.*

## 4. Conclusions

The chemical study of the *Senna cana* and *Senna pendula* ethanol extracts revealed the presence of anthraquinones, flavonoids, tannins, triterpenes, and xanthones, while hexane extracts presented positive results for anthraquinones and triterpenes.

The ethanol extract of the leaves of *S. cana* (LEESC) performed the best in all the antioxidant tests, surpassing the standard values, meaning that this species is a promising source of antioxidants, possibly because of the presence of polyphenolic compounds, especially flavonoids, anthraquinones, and tannins, as confirmed by the quantitative analyses.

The ethanol extract of the branches of *Senna cana* (BEESC) presented the worst performance in all the antioxidant trials, the lowest phenolic content, and the second lowest total flavonoid content. These results confirm that the lower the content of phenolic compounds and flavonoids, the worse the antioxidant capacity of the extract.

This study determined that the hexane extracts presented the best results for acetylcholinesterase activity, which can be attributed to the presence of triterpenes and/or anthraquinones, but the ethanolic extracts of the leaves of *S. cana* (LEESC) presented a satisfactory activity according to the quantitative tests, confirming that the higher the content of phenolic compounds and flavonoids, the better the performance regarding inhibition of acetylcholinesterase.

Most of the extracts investigated showed no toxicity against *Artemia salina*. As stated before, if a sample does not show toxicity to *A. salina*, this means that its effects will also be the same in humans. None of the extracts presented relevant larvicidal activity.

The results obtained in this work indicate that these species are sources of substances with promising pharmacological activities, with emphasis on antioxidant activity, mainly in extracts from the leaves of *Senna cana*.

## Figures and Tables

**Table 1:** Extracts of *Senna* species investigated.

Species	Plant material	Plant dry mass (kg)	Solvent	Abbreviations	Mass extract (g)
*Senna cana* I and B	Leaves	2.165	Hexane	LHESC	20.190
			Ethanol	LEESC	338.010
	Branches	1.950	Hexane	BHESC	15.190
			Ethanol	BEESC	487.001
*Senna pendula* I and B	Leaves	0.932	Hexane	LHESP	24.280
			Ethanol	LEESP	243.110
	Branches	1.498	Hexane	BHESP	27.520
			Ethanol	BEESP	147.790
	Flowers	0.154	Hexane	FHESP	3.780
			Ethanol	FEESP	22.450

LHESC: leaf hexane extract of *Senna cana*; LEESC: leaf ethanol extract of *S. cana*; BHESC: branch hexane extract of *S. cana*; BEESC: branch ethanol extract of *S. cana*; LHESP: leaf hexane extract of *S. pendula*; LEESP: leaf ethanol extract of *S. pendula*; BHESP: branch hexane extract of *S. pendula*; BEESP: branch ethanol extract of *S. pendula*; FHESP: flower hexane extract of *S. pendula*; FEESP: flower ethanol extract of *S. pendula*.

**Table 2:** Phytochemical screening of ethanol extracts of *Senna* species.

Class of the metabolite	LEESC	BEESC	LEESP	BEESP	FEESP
Alkaloids	(−)	(−)	(−)	(−)	(−)
Anthocyanidins	(−)	(−)	(−)	(−)	(−)
Anthraquinones	(+)	(+)	(+)	(+)	(+)
Steroids	(+)	(+)	(+)	(+)	(+)
Flavones	(+)	(+)	(+)	(+)	(+)
Flavonols	(+)	(+)	(+)	(+)	(+)
Saponins	(−)	(−)	(+)	(+)	(+)
Tannins	(+)	(+)	(+)	(+)	(+)
Triterpenoids	(+)	(+)	(+)	(+)	(+)
Xanthones	(+)	(+)	(+)	(+)	(+)

(+): presence; (−): absence.

**Table 3:** Antioxidant activity of *Senna* extracts.

Extracts	DPPH assay (IC50 *µ*g·mL^−1^)^a^	ABTS assay (TEAC)^b^	FRAP assay (EC mg/mL)^c^	ORAC assay (units)^d^
BEESC	87.5 ± 0.01	1398.1 ± 0.03	828.2 ± 0.02	2.06 ± 0.01
LEESC	59.5 ± 0.01	4440.4 ± 0.01	304.8 ± 0.04	4.72 ± 0.03
BEESP	62.1 ± 0.04	4886.7 ± 0.03	329.17 ± 0.01	5.01 ± 0.04
LEESP	70.6 ± 0.02	3963.1 ± 0.09	520.87 ± 0.01	3.83 ± 0.07
FEESP	64.08 ± 0.02	3927.5 ± 0.02	982.56 ± 0.01	2.58 ± 0.01
BHT	350.1 ± 0.03	—	—	—
RUTIN	81.2 ± 0.04	—	485.1 ± 0.07	3.75 ± 0.05

^a^IC_50_: content of the extract able to inhibit 50% of DPPH radicals. ^b^TEAC: antioxidant activity equivalent to trolox (*µ*mol TE. g^−1^ dry weight). ^c^EC: content (mg·mL^−1^) capable of providing an increase in the absorbance reading equivalent to that obtained with a 1 mM solution of Fe(II). ^d^ORAC units represent inhibition of fluorescence quenching induced by 1 mmol·L^−1^ of trolox.

**Table 4:** Correlation between the antioxidant assays.

Antioxidant assay	Correlation
DPPH versus FRAP	*r*=0.8908(*p*=0.0422)(*n*=5)
DPPH versus ORAC	*r*=−0.7453(*p*=0.148)(*n*=5)
DPPH versus ABTS	*r*=−0.95681(*p*=0.011)(*n*=5)
ABTS versus FRAP	*r*=0.79056(*p*=0.111)(*n*=5)
ABTS versus ORAC	*r*=0.8353(*p*=0.078)(*n*=5)
FRAP versus ORAC	*r*=−0.37563(*p*=0.533)(*n*=5)

**Table 5:** Quantification of phenols and flavonoids.

Extracts	TF^a^ (mg·g^−1^)	TPC^b^ (mg of EAG·g^−1^)
BEESC	103.7 ± 0.0045	473.7 ± 0.0233
LEESC	228.9 ± 0.0075	724.5 ± 0.0176
BEESP	87.04 ± 0.0071	541.2 ± 0.0041
LEESP	221.1 ± 0.0077	557.9 ± 0.0063
FEESP	139.7 ± 0.0014	571.6 ± 0.0049

^a^TF (total flavonoid) is expressed in mg of the quercetin·g^−1^ extract. ^b^TPC (total phenolic compound) is expressed in mg gallic acid equivalent per gram of the extract.

**Table 6:** Correlation between the antioxidant assays and total phenolic compounds.

Antioxidant assay	Correlation
DPPH versus total phenolic compounds	*r*=−0.73995(*p*=0.153)(*n*=5)
ABTS versus total phenolic compounds	*r*=0.595982(*p*=0.287)(*n*=5)
FRAP versus total phenolic compounds	*r*=−0.55417(*p*=0.332)(*n*=5)
ORAC versus total phenolic compounds	*r*=0.5769571(*p*=0.308)(*n*=5)

**Table 7:** Correlation between the antioxidant assays and total flavonoid.

Antioxidant assay	Correlation
DPPH versus total flavonoid compounds	*r*=−0.33765(*p*=0.578)(*n*=5)
ABTS versus total flavonoid compounds	*r*=0.29654(*p*=0.628)(*n*=5)
FRAP versus total flavonoid compounds	*r*=−0.1616(*p*=0.791)(*n*=5)
ORAC versus total flavonoid compounds	*r*=0.28349(*p*=0.643)(*n*=5)

**Table 8:** Evaluation of acetylcholinesterase activity of *Senna cana* and *Senna pendula* extracts.

Extracts	Qualitative analysis	Inhibition zone (mm)	Quantitative analysis (IC_50_ *µ*g·mL^−1^)^a^
LEESC	Negative	—	85.5 ± 0.05
BEESC	Negative	—	127.8 ± 0.09
LHESC	Positive	5	80.4 ± 0.001
BHESC	Positive	4	101.5 ± 0.03
LEESP	Positive	6	215.5 ± 0.05
BEESP	Positive	6	106.8 ± 0.06
FEESP	Positive	7	144.0 ± 0.06
LHESP	Positive	8	106.1 ± 0.02
BHESP	Positive	8	195.6 ± 0.002
FHESP	Positive	9	70.3 ± 0.001
Standard (eserin)	Positive	11	19.3 ± 0.05

^a^Acetylcholinesterase inhibition (AChEI) (IC_50_ *µ*g·mL^−1^).

**Table 9:** Results for toxicity against *Artemia salina* of *Senna cana* and *Senna pendula* extracts.

Extracts	LC_50_ (*μ*g·mL^−1^)^a^	Toxicity potential^b^
LHESC	>1000	Nontoxic
BHESC	790.94	Low toxicity
LEESC	>1000	Nontoxic
BEESC	>1000	Nontoxic
LHESP	746.35	Low toxicity
BHESP	>1000	Nontoxic
FHESP	>1000	Nontoxic
LEESP	>1000	Nontoxic
BEESP	>1000	Nontoxic
FEESP	>1000	Nontoxic

^a^LC_50_: lethal concentration to 50% of the larvae of *A. salina*. ^b^Nontoxic: LC_50_ > 1000 *µ*g·mL^−1^; low toxicity: 500 < LC_50_ ≤ 1000 *µ*g·mL^−1^.

**Table 10:** Evaluation of the larvicidal activity of the ethanol extracts of *Senna cana* and *Senna pendula*.

Extracts	LC_50_ (*µ*g·mL^−1^)^a^
LEESC	2182.5
BEESC	1814.8
LEESP	1248.3
BEESP	1271.6
FEESP	918.46

^a^LC_50_: lethal concentration to 50% of the larvae.

## Supplementary Materials

Supplementary Materials This supplementary material is a graphical abstract of this manuscript. This work investigated the total phenols, flavonoids, toxicity, and larvicidal and acetylcholinesterase inhibitory activities and presented the results of four antioxidant assays of the extracts of *Senna* species.
