# Supplementary figures and images for: Bioactivity and Toxicity of Senna cana and Senna pendula Extracts
Source: Biochem Res Int. 2018 Apr 2;2018:8074306. doi: 10.1155/2018/8074306 (PMC5902074; doi:10.1155/2018/8074306)

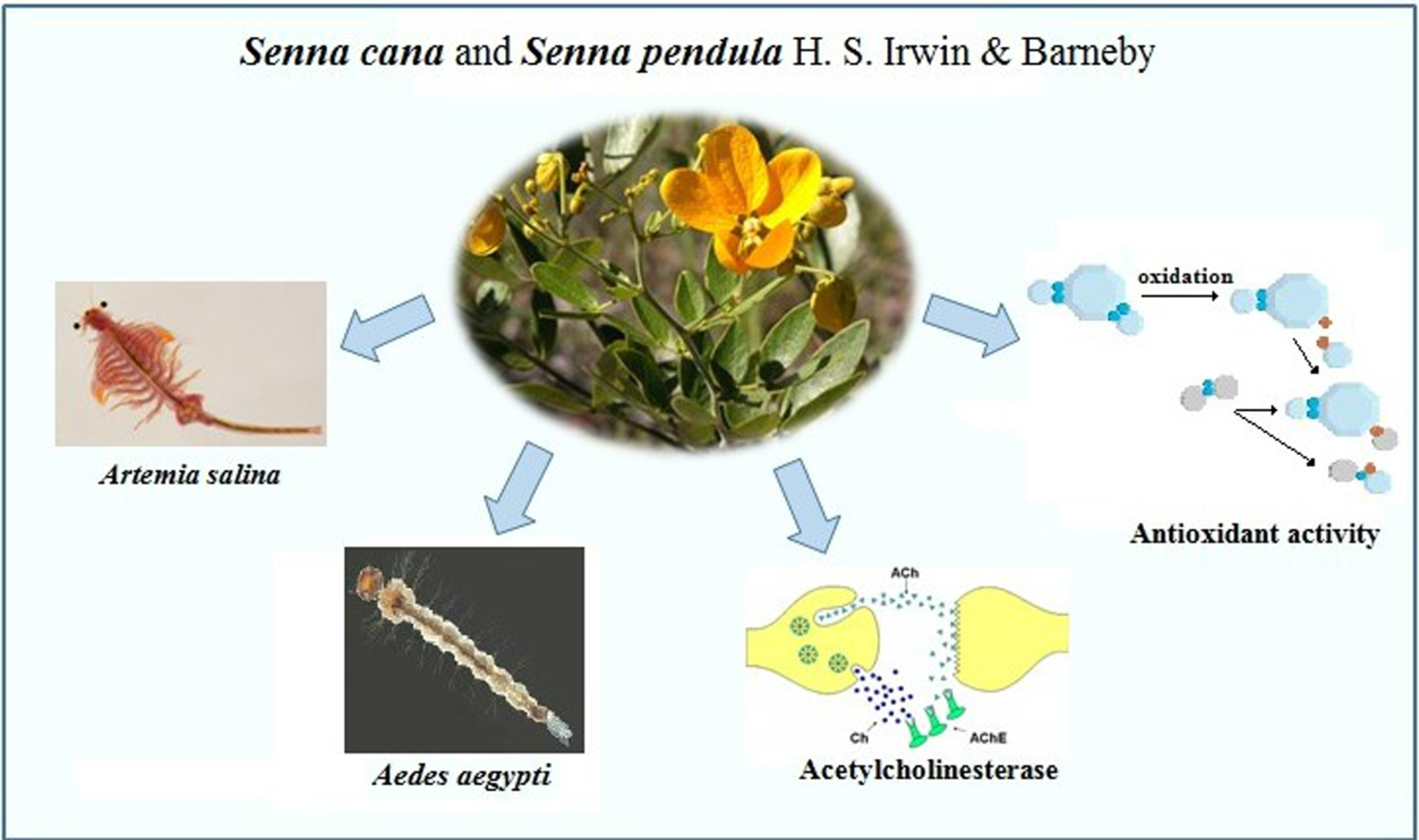

Supplement: Supplementary Materials — This supplementary material is a graphical abstract of this manuscript. This work investigated the total phenols, flavonoids, toxicity, and larvicidal and acetylcholinesterase inhibitory activities and presented the results of four antioxidant assays of the extracts of Senna species. [file 8074306.f1.jpg]
